# Hearing Aid Use and Verbal Fluency as a Measure of Cognitive Function: A Cross-Sectional Study Using SHARE Data

**DOI:** 10.1093/geroni/igaf122.3408

**Published:** 2025-12-31

**Authors:** Meghana Rajashekara Swamy, Adam Mecca

**Affiliations:** Yale University, New Haven, Connecticut, United States; Yale University, New Haven, Connecticut, United States

## Abstract

**Background:**

Hearing aids may mitigate cognitive decline risk. We analyzed its association with verbal fluency, as a surrogate measure for cognition, using the Survey of Health, Ageing, and Retirement in Europe (SHARE) data.

**Methods:**

This cross-sectional study analyzed SHARE Wave 9 (2021–2022) data. Verbal fluency, measured via an animal naming task, was the primary outcome. Self-reported hearing aid use was the primary exposure. Exclusions included dementia, missing data, or age < 50 years. Multivariable linear regression adjusted for demographic, social, and cardiovascular health factors. Analyses used Python 3.9, with α = 0.05 for significance.

**Results:**

After exclusions, 57,433 participants remained. Hearing aid users were older (mean age 76.9 vs. 68.2 years), more often male (50.9% vs. 42.5%), had smaller households (1.77 vs. 2.01), higher net worth (€410,960 vs. €305,221), were less likely to have a household partner (61.2% vs. 68.0%), and reported poorer hearing (28.7% vs. 16.5%). Hearing aid users exhibited higher verbal fluency scores (β = 0.92; 95% CI: 0.18–1.67; p = 0.015). Better hearing level correlated positively with verbal fluency (β = 0.38; p < 0.001), but its interaction with hearing aid use was nonsignificant (p = 0.099). Statistically significant predictors of higher verbal fluency included better social connectedness, education, physical activity, and household net worth, while older age, male gender, stroke, diabetes, hypertension, and larger household size predicted lower performance.

**Conclusion:**

Hearing aid use is linked to better verbal fluency, highlighting opportunities for hearing interventions in cognitive rehabilitation and independence. These findings may reflect reduced cognitive load from treatment, reinforcing hearing loss interventions and expanded hearing aid coverage.

